# The Role of STEM Teaching in Education: An Empirical Study to Enhance Creativity and Computational Thinking

**DOI:** 10.3390/jintelligence13070088

**Published:** 2025-07-18

**Authors:** Suherman Suherman, Tibor Vidákovich, Mujib Mujib, Hidayatulloh Hidayatulloh, Tri Andari, Vera Dewi Susanti

**Affiliations:** 1 Doctoral School of Education, University of Szeged, Petőfi sgt. 30-34, 6720 Szeged, Hungary; 2 Department of Mathematics Education, Universitas Islam Negeri Raden Intan Lampung, Jl. Letkol H. Endro Suratmin Sukarame, Bandar Lampung 35131, Indonesia; mujib@radenintan.ac.id; 3 Institute of Education, University of Szeged, Petőfi sgt. 30-34, 6722 Szeged, Hungary; t.vidakovich@edpsy.u-szeged.hu; 4 Department of Mathematics Education, Universitas Muhammadiyah Pringsewu, Jl. KH. Akhmad Dahlan No.112 Pringsewu Utara, Pringsewu 35373, Indonesia; hidayatulloh@umpri.ac.id; 5 Department of Mathematics Education, Universitas PGRI Madiun, Jl. Setia Budi No.85 Kanigoro, Madiun 63118, Indonesia; triandari.mathedu@unipma.ac.id (T.A.); vera.mathedu@unipma.ac.id (V.D.S.)

**Keywords:** creative thinking, computational thinking, structural equation modelling, secondary education, STEM

## Abstract

This research is focused on exploring the importance of STEM (Science, Technology, Engineering, and Mathematics) education in the development of critical competencies among secondary school students in the 21st century. This was aimed to assess the impact of STEM-based activities on students’ creative and computational thinking skills. A quasi-experimental design that included 77 secondary school students from public and private schools in Bandar Lampung, Indonesia, who participated in STEM interventions for over 5 weeks, was adopted. Data were collected through creative thinking tests and questionnaires on CT and STEM attitudes. The results showed that students who participated in STEM activities exhibited significantly higher creative thinking scores compared to peers taught with the traditional curriculum. Specifically, the experimental group showed a progressive increase in weekly test scores, suggesting that STEM methods improved students’ performance over time. Structural equation modeling (SEM) disclosed strong positive associations between attitudes towards STEM, CT, and creativity. The implications of these results outlined the need to integrate STEM education into curricula to foster essential skills for future challenges. This research contributes to the understanding of effective educational strategies and also advocates for a shift towards more interactive and integrative methods in secondary education to meet the demands of the contemporary workforce.

## 1. Introduction

STEM (Science, Technology, Engineering, and Mathematics) education is deduced to have gained considerable attention globally for its potential to equip students with essential skills for the 21st century (Lin et al. 2023). The integration of STEM in the curriculum aims to provide foundational knowledge and also to foster relevant skills such as problem solving (Tan et al. 2023), creativity (Shahbazloo and Mirzaie 2023), and computational thinking (Wang et al. 2024). Moreover, as the world tends to rely on technology and digital solutions, the ability to think computationally and approach complex problems creatively becomes extremely important.

Creativity, often associated with the arts, plays a crucial role in STEM fields, where innovative thinking leads to groundbreaking discoveries and advancements. STEM teaching has a positive effect on students’ creativity (Aguilera and Ortiz-Revilla 2021), including the development process (Wang and Li 2022). Meanwhile, computational thinking (CT), a systematic approach to problem solving that entails pattern recognition, abstraction, algorithmic thinking, and decomposition (Suherman and Vidákovich 2024b), enables students to tackle complex issues effectively. As a predictor of STEM learning (Jiang et al. 2024), related courses were taken to improve learners in plugged CT activities (Galanti and Holincheck 2024). Technical skills in CT are also essential for solving problems, including possession of the appropriate attitude. When students approach learning and problem solving with curiosity and positivity, CT can be used more effectively to tackle real-life challenges (Hamutoğlu et al. 2022). As a result, a favorable attitude toward CT fosters collaboration among peers. It also enabled students to work in teams, share diverse perspectives, and use collective strengths to solve problems, increasingly relevant in globalized contexts (Nouri et al. 2020). The attitudes exhibited towards these skills played a significant role in overall effectiveness and application, as well as fostered positive attitudes, namely, resilience, creativity, and collaboration, including commitment to lifelong learning. Educators aim to develop well-rounded students who can navigate, adapt, and contribute to the rapidly changing technological landscapes, by integrating STEM education that outlines these skills. However, the analysis on attitudes toward CT is rare, including those outlining the novelty of this present research.

In this context, the attitude of students toward STEM is essential to optimize learning outcomes, particularly in developing CT skills. A robust attitude serves as a precursor for successful engagement in STEM teaching strategies, which vary significantly depending on educational contexts. Some research (Boeve-De Pauw et al. 2024; Goos et al. 2023; Maskur et al. 2022) stated that the implementation of these strategies was highly influenced by factors such as curriculum design, teacher training, and the availability of educational resources. Additionally, Ernst et al. (2018) reported the existence of considerable disparity in the understanding and application of STEM competencies across various global educational systems. This implied that positive attitudes were crucial for students to navigate the variations. Based on the description, while the qualifications in various related fields, including mathematics, chemistry, computer science, biology, physics, architecture, and engineering disciplines were properly defined (Stoet and Geary 2018), the interpretations of STEM constituents differed significantly (Fitzakerley et al. 2013). The complexity outlined the need for educators to foster a constructive STEM attitude among students to improve confidence and competency in applying related knowledge in diverse fields.

The meta-analysis conducted by Cheng et al. (2023) showed that integrating STEM learning with CT in middle school environments substantially improved students’ problem-solving skills and interdisciplinary understanding. This depicted that the cultivation of a positive attitude synergistically supported students in developing essential computational thinking capabilities during engagement with complex interdisciplinary challenges. However, the research by Maskur et al. (2022) reinforced the premise that a supportive and positive learning environment was critical, as it collectively enhanced students’ attitudes and competencies. In view of these results, fostering a robust STEM attitude was an essential requirement for students, influencing both engagement with the teaching strategies and overall success in developing computational thinking skills. This prepared students for future academic and professional accomplishments.

This research explored the implementation of teaching practices specifically designed to improve creativity skills, STEM, and CT attitudes among students. Following the description, creativity skills, including STEM and CT attitudes, were the two critical competencies for the 21st century. Although existing research has explored the positive effects of STEM education on creativity, including the role of CT as a predictor of success in the learning process, there are limited reviews on how STEM instructional methods simultaneously foster these two interconnected skills and attitudes. This present research addressed the gap by analyzing specific teaching strategies and their effectiveness in diverse educational contexts. The main objective was to evaluate the impact of STEM-focused activities on students’ creative thinking and CT skills. This enabled the contribution to the growing body of the literature on STEM education by providing insight into the practical applications of innovative teaching methods and the potential to shape well-rounded and future-ready individuals. In addition, the following hypotheses were formulated:

**H1.**  *The creative thinking and CT assessment tool exhibited satisfactory psychometric properties, confirming its validity and reliability.*

**H2.**  *Creative thinking had a positive impact on CT.*

**H3.**  *STEM attitude had an impact on students’ CT.*

**H4.**  *Creative thinking served as a mediating factor in the pathway between STEM attitude and CT.*

### 1.1. Creative Thinking and CT

Creative thinking is the ability to actively participate in formulating, assessing, and refining ideas, leading to unique and effective solutions, knowledge development, and meaningful imaginative expressions (OECD 2019). At the same time, the ability to generate novel and valuable ideas is a crucial skill for problem solving and innovation (De Jager et al. 2013; Suherman and Vidákovich 2022). This includes the capacity to view situations from multiple perspectives, challenge assumptions, and explore unconventional solutions (Torrance 1974). Creative thinking has been widely recognized as an essential component of learning, which fosters flexibility, adaptability, and critical engagement with content (Suherman and Vidákovich 2022). The pioneering research by Guilford (1950) described creativity as a special talent and mental ability nurtured and developed through practice in the appropriate environment. Based on the education context, the development of creative thinking motivated students to deeply engage with learning materials, facilitating personal expression and improving overall cognitive growth.

Recent research focused on the role of creative thinking in various disciplines. For example, Cromwell et al. (2023) stated that associating thinking styles (i.e., creative thinking) with the task structure improved overall motivation and performance. Additionally, Besançon and Lubart (2008) reported that students exposed to creative learning environments performed better in problem-solving tasks due to the ability to think divergently and explore multiple solutions. The integration of creative thinking into teaching also prepared students for complex real-world challenges, as it enabled the development of skills essential for adapting to the rapidly changing job markets (OECD 2019). Meanwhile, various strategies, such as open-ended tasks, brainstorming sessions, and interdisciplinary projects, have been proven to effectively foster creative thinking in students, particularly when educators motivate risk taking and experimentation (Sawyer 2012). These results show the importance of embedding creative thinking practices in educational settings to cultivate innovative and adaptable intellectuals.

Empirical research has shown that creativity significantly enhances CT by promoting problem-solving flexibility and innovative reasoning. For example, research by Hershkovitz et al. (2019) reported that a relationship existed between creative thinking and CT. This implied that originality in tasks was connected to success during the initial stages but negatively associated with further progression, outlining the dynamic relationship between these constructs. Xu et al. (2022) reported that fostering creativity through open-ended STEM activities led to significant engagement and better performance in computational problem solving, outlining the interconnected nature of the cognitive skills. The research on young children (aged five to six years) showed that CT directly supported creative thinking. The result proved that CT skills positively influenced the development of creativity, with arithmetic fluency acting as a mediator in its relationship with reasoning ability.

### 1.2. Computational Thinking

CT is a problem-solving process comprising a set of skills and approaches drawn from computer science. It enabled individuals to tackle complex problems across various disciplines (Hsu et al. 2018). Previous research outlined the importance of integrating CT into educational curricula, with reviews showing the effectiveness in enhancing students’ problem-solving skills and understanding of core concepts in various subjects. The incorporation of computational modeling and programming into K-12 science and mathematics curricula could be difficult. This was due to the significant demands on teachers and hurdles encountered by students when learning programming (Sengupta et al. 2013; Sherin et al. 1993). Furthermore, Weintrop et al. (2016) and Lee and Malyn-Smith (2020) designed frameworks aimed at integrating computational thinking practices in STEM disciplines in grades K-12. The significance of CT in education has attracted considerable interest from diverse research, resulting in a trend toward its incorporation into school curricula (Angeli et al. 2016; Wing 2006). Many educational institutions are currently reviewing respective computer science programs to outline fundamental concepts and principles.

Considering that the technical skills of CT are crucial to solving problems through systematic and algorithmic methods, the role of attitude is equally important but often overlooked. The possession of a positive attitude toward research and learning can make a significant difference in the proper application of CT (Hamutoğlu et al. 2022). In simpler terms, CT refers to a blend of knowledge, skills, and attitudes that allow the use of computers to solve real-world problems, obtaining meaningful outcomes (Korkmaz et al. 2017). In this context, CT attitude is increasingly crucial in navigating the challenges of the 21st century. It also plays a significant role in equipping students for a rapidly evolving technological landscape. According to Yadav et al. (2022), having a positive attitude toward the development of CT competencies in education is crucial, specifically in a digitalized democratic society. The research by Budhi Akbar (2022) focused on problem-based learning in biology classes and found that students’ social attitudes, namely, cooperation, tolerance, and confidence, correlated positively with respective CT. The results suggested that the cultivation of a supportive social environment enhanced the problem-solving capabilities of students through CT. Simultaneously, those with positive attitudes toward programming also exhibited enhanced CT skills and higher levels of motivation. This interconnectedness focused on the relevance of cultivating a positive attitude towards CT from an early age, particularly in primary education, where foundational skills were developed (Richardo et al. 2023). Additionally, the research proved that positive attitudes towards CT significantly impacted students’ problem-solving skills. This depicted that educators needed to focus on cultivating the attitudes alongside cognitive skills (Lucas et al. 2013). Previous research reported how the attitudes could be integrated into CT education. Students’ attitudes were proven to influence respective CT development, making it a significant factor in enhancing related skills (Tikva and Tambouris 2023). In line with this present research, there is a need to consider the attitudes of students toward CT. Furthermore, positive attitudes greatly improved students’ beliefs and perceptions (Fessakis and Prantsoudi 2019).

### 1.3. STEM Education and CT

STEM refers to an interdisciplinary educational approach that combines rigorous academic concepts with practical applications in the real world (US STEM Task Force 2014). In recent years, it has received significant attention for its ability to prepare students with the relevant skills for success in a rapidly changing technological environment (Ouyang and Xu 2024). Prior research reported that students benefitted more from learning when multiple STEM disciplines were integrated into a single class (Kelley and Knowles 2016). This integration fostered knowledge construction by immersing students in technology- and engineering-based learning experiences. The focus on investigation and solution of real-world problems enabled the motivation of students to explore and enquire about the surroundings (Acar et al. 2018).

Various research studies have reported that hands-on learning experiences, such as project-based learning and inquiry-based activities, significantly improved students’ engagement and motivation in STEM subjects, leading to higher academic performance and increased interest in pursuing related careers (Chen et al. 2023; Wiswall et al. 2014). Furthermore, the implementation of STEM education in schools often faced the following challenges: insufficient teacher training, lack of resources, and limited curriculum alignment (Lesseig et al. 2016; Lo 2021; Makhmasi et al. 2012). The need for equity was also outlined, as under-represented groups encountered systemic barriers to accessing quality STEM learning opportunities. Addressing these disparities was crucial to foster a diverse and inclusive workforce that drove innovation and problem solving in the 21st century. Generally, the research outlined the transformative potential of STEM education in preparing students for future challenges and opportunities in a globalized, technology-driven world.

STEM education centered the need for students to acquire multidisciplinary knowledge, including science, technology, engineering, and mathematics, as well as tackling real-world, open-ended, and poorly defined problems (Ouyang and Xu 2024). In secondary schools, STEM attitude in education was delivered through project-based learning, integrated curriculum design, and hands-on activities that promoted critical thinking, collaboration, and problem solving (Maskur et al. 2022). For example, students might work in teams to design a bridge using engineering principles, develop a coding project to solve a local community problem, or apply mathematical modeling to analyze scientific data.

Following the description, STEM attitudes played a crucial role in shaping students’ engagement with CT and overall performance in related tasks. Preliminary research has shown that students who hold positive attitudes towards STEM education exhibited stronger CT skills (Sun et al. 2021), as the enthusiasm and confidence in the subject matter motivated greater participation in problem solving and analytical tasks. For example, Sun et al. (2021) stated that students’ learning attitudes toward STEM directly predicted CT skills, thereby reinforcing the role of a supportive educational environment in nurturing both interest and competency in these essential areas. The general trends in educational research support the inference that interventions aimed at enhancing positive attitudes toward STEM, such as inquiry-based learning and out-of-school programs, substantially improved engagement and academic performance in CT (Baran Jovanovic et al. 2019; Psycharis and Kotzampasaki 2019). Verawati et al. (2023) reported that the integration of technology in STEM practices supports participation and enriches the educational landscape, significantly improving the CT skills of students. The research by Richardo et al. (2023) also reported a strong correlation between STEM attitudes and CT, implying that the cultivation of positive emotional connections to related fields improved the computational skills of students. However, Chiang et al. (2022) stated that participation in online STEM camps significantly improved the CT of students, exhibiting the positive impact of STEM education on enhancing this critical skill.

### 1.4. Creative Thinking Mediates STEM Education and CT

Creative thinking played a crucial mediating role in the relationship between STEM education/attitude and CT. In STEM education, teachers migrate from traditional lecturing to acting as facilitators, motivating students to explore and become innovative. The assessment methods also reflected this shift, focusing on creativity, communication, and teamwork, rather than just content knowledge. However, traditional teacher-centered approaches that prioritized knowledge delivery and rote memorization hindered the development of creative thinking and critical computational skills, affecting students’ overall learning outcomes in STEM (Xu and Ouyang 2022).

**Figure 1:**
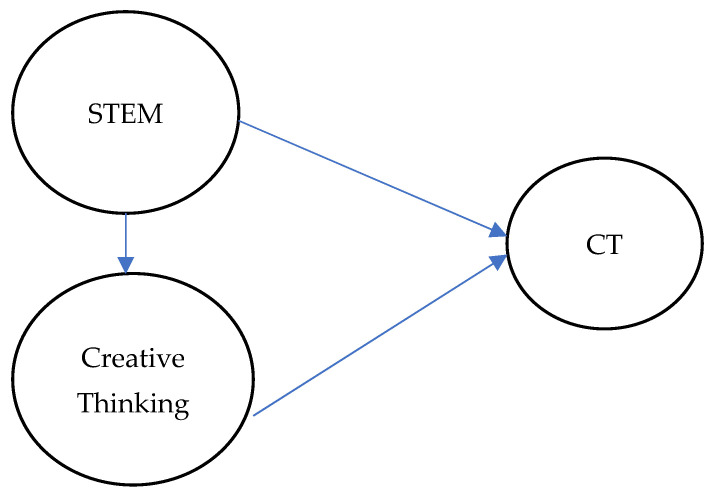
Theoretical model.

Empirical research supported the idea that creative thinking acted as a mediator between STEM education and CT. A quasi-experimental study found that STEM-based scientific learning significantly improved students’ critical and creative thinking compared to conventional methods. In addition, statistical analyses confirmed that creative thinking served as a predictive factor for these outcomes (Astawan et al. 2023). Longitudinal research also reported that integrating creativity into STEM curricula strengthened scientific problem-finding skills and fostered positive attitudes toward STEM careers, despite the persistent gaps in sustained engagement at higher educational levels (Pont-Niclòs et al. 2024). This mediation was supported by a specific research carried out in Malta, which reported STEM exposure and enjoyment positively correlated with divergent thinking performance, even after monitoring variables such as age and parental education (Borg Preca et al. 2023). Collectively, the numerous studies perceived creativity as a fundamental mechanism through which STEM education cultivated deeper cognitive skills and positive disciplinary attitudes. Based on this view, the theoretical model is shown in Figure 1.

## 2. Materials and Methods

### 2.1. Participants

Participants comprised 77 secondary school students (54.5% female, 45.5% male) with an average age of 12.70 years (*SD* = 0.61), representing both public and private schools in Bandar Lampung City, Indonesia. The student sample was randomly selected from the intended schools based on the integration of STEM curriculum and accessibility. The selection process was carried out through a simple random sampling technique, to ensure that all students were exposed to similar chances of being included. The final sample consisted of students mainly from Java (71.4%). Both institutional and school approval were obtained prior to this research, with all students providing informed consent to participate. In addition, the demographic details of participants are shown in Table 1.

### 2.2. Research Procedures

Data collection methods consisted of documentation and testing, with instruments that included a creative thinking test and a questionnaire used to evaluate attitudes towards CT and STEM. Prior to detailed hypothesis testing, an initial assessment of the mathematical creativity and CT skills of students was carried out in each treatment group to verify certain conditions. The results of the pre-test confirmed that there were no significant differences between the groups. The baseline mathematical creativity and CT scores, t(71) = 1.37, *p* = 0.175, Cohen’s d = 0.16, ensured comparability. Additionally, normality tests and validity checks of the instruments were performed to confirm the suitability of the assessments. Participants were boys and girls aged 12 and 14 who met specific initial diagnostic criteria, such as class normality testing. This research did not exclude participants according to other social science criteria, making it inclusive of eligible community members. Ethical guidelines and permissions for data collection were established through the institutional review board (IRB) from Universitas Islam Negeri Raden Intan Lampung, based on the permission of the school.

### 2.3. Research Design

This research used a quasi-experimental post-test-only design, following an initial teaching intervention, including a post-test that measured students’ creative thinking skills. STEM-based activities were assigned to the experimental group 3 days a week from 23 August to 27 November 2023, with the results of the post-test for creative skills collected from both the experimental and control groups. Table 2 shows a typical example of the experiment activities.

The inclusion criteria specified that only grade 8 students were selected, due to the categorization of these students in the developmental stage characterized by the ability to engage in higher-order thinking skills, including problem solving and CT. In addition, students at this level were typically exposed to basic STEM concepts, which enabled the suitable assessment of STEM attitudes on CT. The selection process also ensured consistency in cognitive and educational background, thereby reducing potential confounding variables that could arise from differing grade levels.

**Figure 2:**
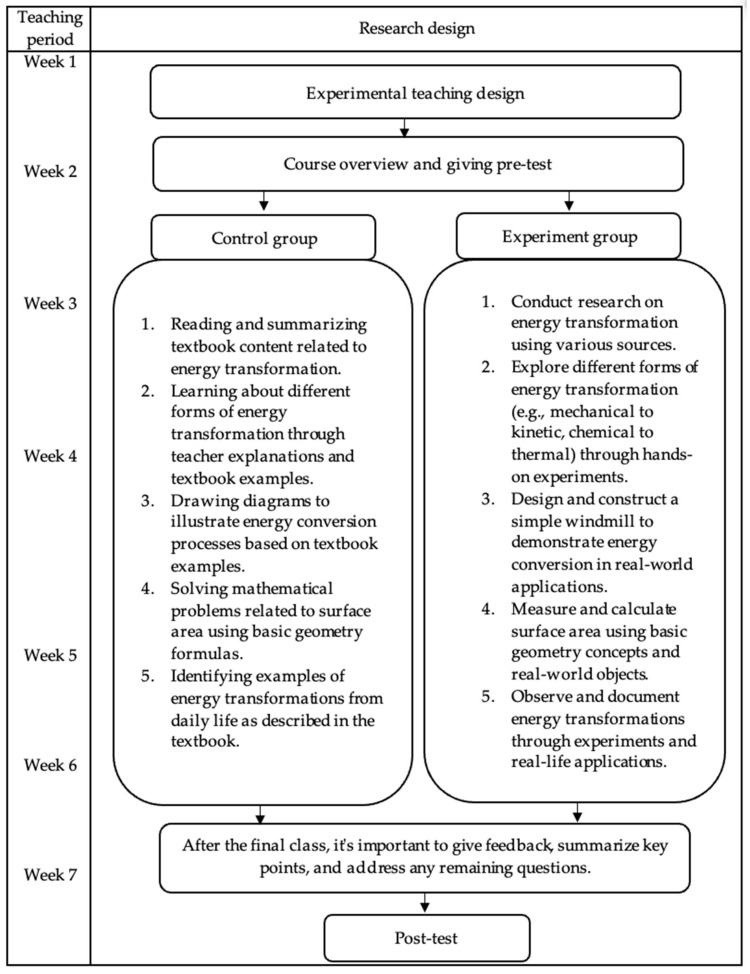
Syntax teaching.

The instructional approach relied on small group activities, with students organized by age, interest, developmental stages, and skill levels. In these groups, students collaborated when designing and marketing products to peers, an approach proven to be more effective than one-on-one instruction (Huda et al. 2019; Suherman et al. 2021; Yasin et al. 2020). However, the control class followed the standard curriculum implemented by its teachers, which included traditional teaching methods in line with existing educational standards. The curriculum mainly focused on lecture-based instruction, individual assignments, and a limited outline on active, hands-on problem-solving strategies. It lacked additional STEM-based activities, serving as a benchmark for comparison. The experimental design adopted is shown in Figure 2.

The activities for each day resumed around 7:30 a.m. and lasted for approximately 60 to 90 min, starting with a physical task to make the students energetic. After completion of the 5-week STEM program, the creative thinking skills of students were evaluated, while the results of the experimental and control groups were compared. Weekly tests were also given, and the evaluation process designed to assess students’ understanding of the topics covered, with specific content varying accordingly. The assessments consisted of a set of questions in line with the material for that week and were not identical with the subsequent week. Regarding this perspective, the scoring system ranged from 0 to 100.

A follow-up questionnaire on STEM attitudes and CT was distributed to evaluate the understanding and comfort of students with the skills. To ensure a relaxed atmosphere, the questionnaires were completed individually in teachers’ lounge, with each session lasting 20–25 min. This setup allowed students to express respective competencies in multiple ways, ensuring a well-rounded assessment (Henriksen et al. 2016).

### 2.4. Instruments

STEM attitude was measured using a four-item scale adapted from Jiang et al. (2024). Sample elements included statements such as If I learn engineering, then I can improve equipment and tools used daily; I am good at building and fixing structures; Designing products or structures will be important for my future work; and I am curious about how electronics work. The responses were rated on a five-point Likert scale, from 1 (strongly disagree) to 5 (strongly agree). Additionally, the validity and reliability of the scale were also assessed.

In view of the description, CT was measured through students’ attitudes. Based on this perspective, the attitude towards CT was evaluated using a four-item scale adapted from Korkmaz et al. (2017). Examples of CT items included statements such as I trust that I could formulate a plan and use it to solve any problem; I can mathematically express solutions of problems faced daily; I am willing to learn challenging issues; and I can produce many options while thinking about possible solutions to a problem. The responses were rated on a five-point Likert scale, from 1 (never) to 5 (always). Furthermore, the reliability and validity of the instrument were also evaluated.

Suherman and Vidákovich (2024a) stated that creativity was evaluated through an essay test consisting of four items that covered the following components: flexibility, fluency, elaboration, and originality. The responses were classified according to predefined answer types, with a rating of 5 depicting the highest scores for flexibility and fluency. Originality scores were calculated using percentage ranges, such as scores above 3% were given a value of 0, between 2% and 3% received a score of 1, between 1% and 2% were rated 2, and those below 1% were assigned a 3. In respect to this view, the elaboration was scored as 1 or 2, with the reliability and validity of the instrument subsequently measured.

**Figure 3:**
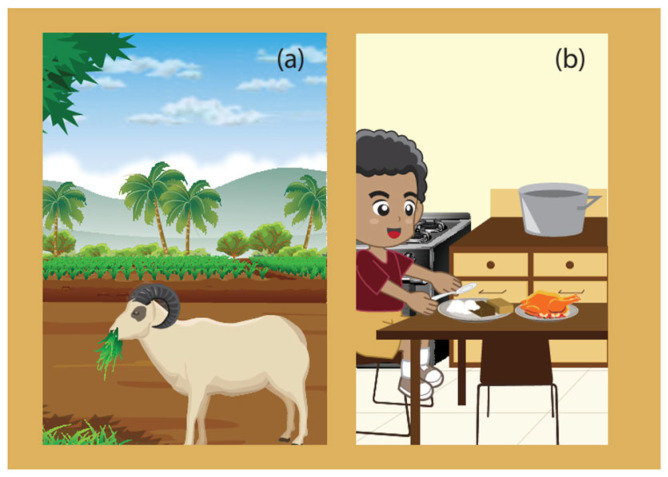
Energy consumption (Ministry of Education 2014). See the figure above: (**a**) the goat eats grass; (**b**) the human eats rice. Based on the figure above, answer the following questions: 1. How do individuals creatively solve problems related to energy consumption in daily appliances, considering the understanding of energy transformations? 2. In what ways do creative approaches to managing energy use in household appliances impact daily routines and overall energy efficiency?

The weekly assessment was designed to evaluate students’ activities during teaching and learning. It comprised five essay items, with an item delivered each week, as shown in Figure 3.

### 2.5. Data Analysis

This research used SPSS 29 to carry out descriptive statistical analyses and correlation of variables. Furthermore, SmartPLS 4 was adopted to analyze all variables using structural equation modeling (SEM), alongside the validity and reliability of the constructs assessed with confirmatory factor analysis (CFA). The factor loading of each item exceeded 0.40, meeting the acceptable threshold. Composite reliability (CR) and Average Variance Extracted (AVE) values must exceed 0.70 and 0.50, respectively, following the criteria of Fornell and Larcker (1981). Discriminant validity was assessed using the heterotrait-monotrait ratio (HTMT), with a threshold of 0.90 considered acceptable (Kline 2015). In terms of data normality, Kline (2015) suggested that skewness should not exceed |3|, and kurtosis must be less than |10|.

Model fit was evaluated using multiple indices, including chi-square statistics (with degrees of freedom and *p*-values), Comparative Fit Index (CFI), Tucker–Lewis Index (TLI), Root Mean Square Error of Approximation (RMSEA), Goodness of Fit Index (GFI), and Standardized Root Mean Square Residual (SRMR) (Kline 2015). CFI value greater than 0.90 depicted an acceptable model fit. RMSEA was interpreted in a manner that values greater than 0.08 depicted poor fit, and values of 0.08 or less than suggested good fit. SRMR also served as an absolute fit index to assess the adequacy of the model. Furthermore, factor loadings greater than 0.80 were considered statistically significant.

The simple one-way Analysis of Variance (ANOVA) was adopted to compare STEM and control groups after each week. Additionally, the R software version 4.4.1 was used to create visual representations of the relationships between the scale variables, providing insight into students’ performance.

## 3. Results

This section was divided into subheadings, providing a concise and precise description of the experimental results. Additionally, the obtained results were interpreted, and the experimental conclusions drawn.

### 3.1. Construct Reliability and Validity

Table 3 shows the loading factors, reliability, and validity of the data for three measured constructs, namely, CT, creative thinking, and attitude towards STEM. The outer loadings for each item in the constructs showed strong associations with the respective latent variables, with most loadings exceeding the 0.05% threshold, reflecting good item reliability. CT achieved Cronbach’s alpha and a composite reliability of 0.821 and 0.881, respectively, suggesting adequate internal consistency, with an AVE of 0.651, implying good convergent validity. Additionally, creative thinking exhibited satisfactory reliability (Cronbach’s alpha and composite reliability of 0.777 and 0.852, respectively), with an AVE of 0.593. The attitude towards STEM showed high reliability, with Cronbach’s alpha, composite reliability, and AVE of 0.920, 0.943, and 0.806, respectively, suggesting strong internal consistency and convergence validity.

In terms of data normality, Kline (2015) stated that skewness values should not exceed |3|, and kurtosis must be less than |10|. For this research, the skewness values ranged from −0.15 to 0.09, while kurtosis was observed in −0.29 and 0.34.

### 3.2. Discriminant Validity

Table 4 shows the discriminant validity of the constructs evaluated using the heterotrait-monotrait (HTMT) ratio. HTMT values of the constructs were less than the generally accepted threshold of 0.90, implying that the constructs were significantly distinct from each other. Specifically, the HTMT ratio between creative thinking and CT was 0.758. The value of the ratio between creative thinking and attitude toward STEM was 0.537. The relationship between computational thinking and attitude towards STEM was 0.848. These values suggested adequate discriminant validity, confirming that each construct measured a unique concept.

### 3.3. Statistical and Descriptive Weekly Assessment

A Mann–Whitney U test was conducted to compare the pre-test scores between the experimental and control groups. The results showed that there was no significant difference in pre-test scores between the two groups (*U* = 685.00, Z = −0.579, *p* = 0.562). This suggested that both groups had comparable baseline scores before the treatment was applied.

Table 5 shows the results of the weekly evaluation for both students in experimental (STEM) and control classes over a 5-week period. The experimental group, which adopted STEM-based teaching methods, showed a progressive increase in weekly test scores, starting from an average range of approximately 47.22 in week 1 and reaching relatively 77.41 by week 5. This upward trend suggested that STEM teaching methods may positively impact students’ performance over time.

The control class, which did not follow a STEM-based approach, showed improvement, but at a slower pace, with average scores of approximately 41.22 in week 1, rising to 66.70 by week 5. Although improvement was observed in both groups, the experimental group consistently achieved higher scores than the control each week, showing that STEM-based teaching methods were more effective in improving students’ weekly test performance over the course of investigation.

Table 6 shows the descriptive statistics for the three variables—CR, CT, and ST—in both the control and STEM groups. In the control group, the mean scores for CR, CT, and ST were 3.17, 3.17, and 3.30, respectively, with standard deviations ranging from 0.64 to 0.93. The minimum and maximum values for these variables were between 1.00 and 5.00, showing a moderate range of responses. In the STEM group, the mean scores for CR, CT, and ST were slightly lower, with averages of 3.06, 3.05, and 3.15, respectively, and standard deviations ranging from 0.72 to 1.12. Similar to the control group, the minimum and maximum values ranged from 1.00 to 5.00, showing that the responses covered the full scale.

The results of ANOVA were obtained using the following step, which helped to ascertain whether there were differences between the groups at each point in time. The simple ANOVA was selected because it was easy to understand and clearly showed group differences weekly. In Week 1, the difference between groups was statistically significant (*F*(1,10) = 8.01, *p* < .05, $\eta_{p}^{2}=0.45)$, suggesting that the STEM group performed better than the control. However, in Week 2, the difference was not statistically significant (*F*(1,10) = 4.73, *p* < .05, $\eta_{p}^{2}=0.32$), showing that the two groups had relatively similar performance levels during this period. By Week 3, the performance gap widened, with a significant difference (*F*(1,10) = 12.87, *p* < .05, $\eta_{p}^{2}=0.56$), showing that STEM group was consistently improving at a faster rate. The most significant differences were observed at weeks 4 and 5, where STEM group exhibited a significantly higher performance than the control group. In Week 4, this difference was statistically significant (*F*(1,10) = 88.63, *p* < 0.001, $\eta_{p}^{2}=0.89$), and the gap widened even further in Week 5, with an even stronger effect (*F*(1,10) = 371.49, *p* < 0.001, $\eta_{p}^{2}=0.97$). The results suggested the substantial impact of STEM approach on students’ performance, particularly in the later weeks of the analysis.

Repeated measures using ANOVA were aimed at investigating the effect of the teaching method (STEM vs. control) on students, observed by the weekly test scores for over 5 weeks. The within-subject analysis showed a significant effect of time on both groups. For the control group, there was a statistically significant change in test scores across the weeks, *F*(2.355, 89.491) = 64,907,571.3, *p* < .001, $\eta_{p}^{2}=0.85$. Similarly, the experimental group also showed a significant variation over time, *F*(3.605, 133.693) = 408,422.75, *p* < .001, $\eta_{p}^{2}=0.92$. Mauchly’s sphericity test implied that the assumption of sphericity was violated in both groups, *W* = 0.273, *p* < .001, and *W* = 0.769, *p* < .05, for the control and experimental groups, respectively. These violations suggested that the variance of the differences between repeated measures was not equal, and appropriate corrections (e.g., Greenhouse–Geisser) were applied. Generally, the results showed that students in both groups experienced significant changes in performance over time, with the STEM group showing a more pronounced improvement.

Table 6 shows the correlation coefficients between the variables for both groups. In the control group, creativity moderately correlated with CT (*r* = 0.654, *p* < .01) and had a weaker correlation with the attitude towards STEM (*r* = 0.450, *p* < .01). Meanwhile, CT in the control group strongly correlated with STEM attitude (*r* = 0.732, *p* < .01). For the STEM group, a similar pattern was observed, with creativity and CT showing a moderate positive correlation (*r* = 0.576, *p* < .01) and CT observed to be highly correlated with STEM attitude (*r* = 0.742, *p* < .01).

### 3.4. SEM Evaluation

**Figure 4:**
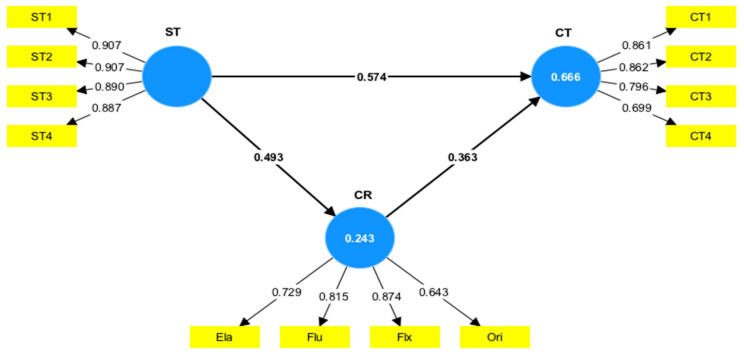
SEM model.

During the last session, students were given a questionnaire and test. The questionnaire aimed to assess respective attitudes during classes in both STEM and control teaching models. SEM analysis was conducted to examine whether the STEM teaching model was influenced by creativity and CT. Based on Figure 4, which summarized the standardized relationships among variables, the fit indices of the model were reported as follows: chi-square = 124.961, df = 51, *p* < .001, CFI = 0.93, TLI = 0.91, RMSEA = 0.08, and SRMR = 0.07. As stated previously, the recommended threshold for CFI and TLI was >0.90, while RMSEA and SRMR should be <0.08 (Hu and Bentler 1999; Kwong-Kay Wong 2013; Meyers et al. 2016). These results showed that the model had an acceptable fit, meeting the criteria suggested for the CFI, TLI, RMSEA, and SRMR.

In terms of coefficient determination, STEM represented 66.6% and 24.3% of the variance in CT (R^2^ = 0.666) and creativity (R^2^ = 0.243), respectively. Regarding the path coefficients, the attitude of students toward STEM showed a strong positive association with both CT (*β* = 0.574, *p* < .001) and creativity (*β* = 0.493, *p* < .001). Furthermore, creativity was positively associated with CT (*β* = 0.363, *p* < .001), outlining the interconnected relationships between these variables. A post hoc power analysis confirmed sufficient statistical power of 0.95 to detect medium to large effects at *α* = 0.05. This research addressed the main conceptual gap by integrating affective and cognitive constructs in a single model, offering a novel perspective on how students’ attitudes in STEM classrooms fostered both creativity and CT essential for 21st-century learning.

Table 7 shows the direct and indirect effects of the variables. However, a bootstrapping method was applied with 5000 iterations to evaluate the mediating role of creativity in the relationship between CT and students’ attitudes toward STEM. The results implied that creativity significantly mediated this relationship, showing a positive association between CT and STEM attitude (*β* = 0.179, *p* < .001).

**Figure 5:**
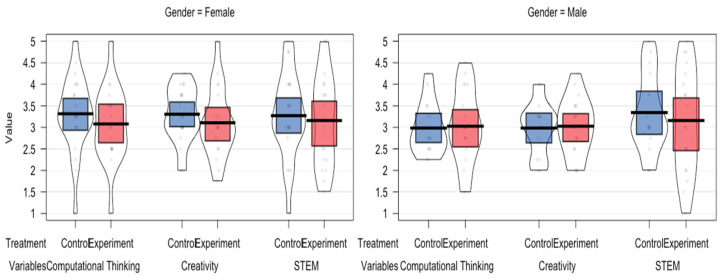
Plot between control and experiment groups among genders in different skills.

**Figure 6:**
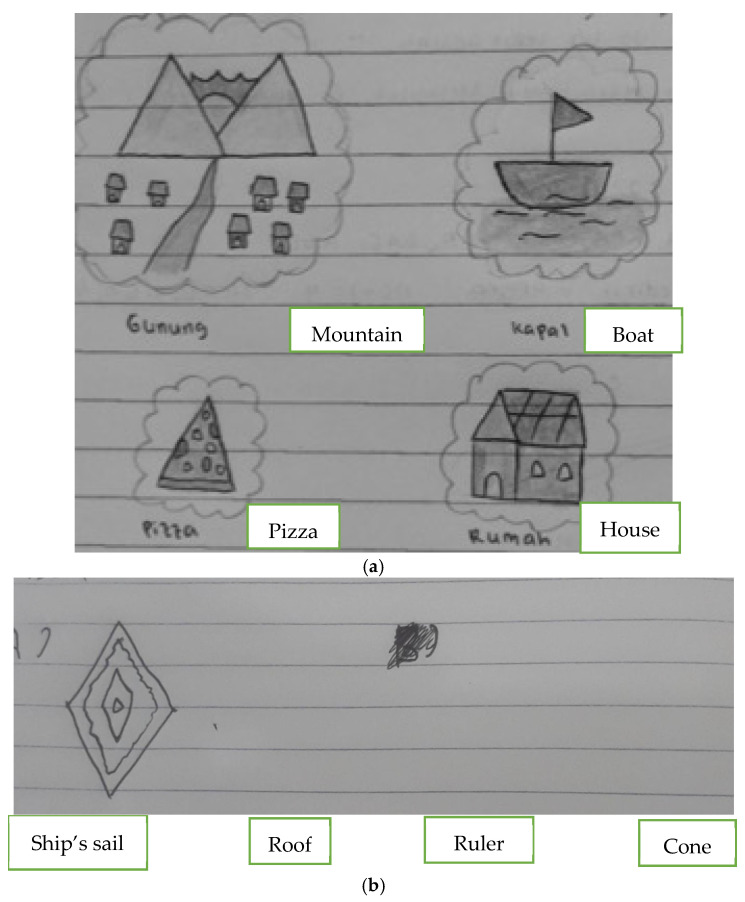
Students’ answer in (**a**) the experimental class (STEM) and (**b**) the control class.

The statistical descriptiveness is shown in Figure 5. Additionally, students’ answers in both the experimental and control classes are also shown in Figure 6.

## 4. Discussion

The results of the research showed that the tools/instruments used were both valid and reliable. This outlined the importance of ensuring that the measurement tools were accurate and consistent in assessing complex constructs such as creativity and STEM-related attitudes. For example, research by Suherman and Vidákovich (2024a) reported that valid and reliable instruments were essential for capturing the multidimensional nature of creative thinking. Jiang et al. (2024) and Korkmaz et al. (2017) outlined the need for reliable CT and STEM attitude tools to gauge students’ engagement in these fields. The validity and reliability of the instruments played a crucial role in obtaining meaningful and actionable insights.

Based on the description, the instruments used were both valid and reliable. Previous research had reported the importance of using reliable and valid instruments to assess complex constructs such as creativity and STEM engagement. For example, Beghetto and Kaufman (2014) stated that reliable tools were essential for measuring creative thinking accurately. Guzey et al. (2014) reported that valid STEM attitude instruments helped assess students’ engagement and motivation in these fields. Therefore, the results confirmed the effectiveness of the instruments in capturing the main aspects of the cognitive and attitudinal development of students.

The results outlined the positive impact of STEM-based education on improving students’ creativity and CT skills. This suggested that integrating STEM methods fostered essential skills required for problem solving and innovation in educational settings. The results were in line with previous research (Borg Preca et al. 2023; Sun et al. 2021), which reported the positive influence of STEM-based education on students’ creative and CT skills, mainly due to the integration with information technology. By combining technological tools with mathematical reasoning and techniques aimed at fostering idea generation, it was found that STEM education could effectively guide students through the problem-solving process.

STEM education further equipped students with greater awareness of real-world issues, enabling the formulation and justification of diverse solutions to daily phenomena that require critical thinking. Although resources for information retrieval were limited, students exposed to STEM learning developed stronger creative and computational skills, resulting in thoughtfully and innovative engagement with complex challenges in the surroundings.

The experimental classroom, using STEM-based teaching methods, showed a significant increase in weekly assessment scores as students engaged in hands-on, interdisciplinary activities that integrated STEM. This method was in line with previous research, which found STEM learning improved students’ engagement and retention by connecting academic concepts with real-world applications (Struyf et al. 2019), motivating critical thinking and problem-solving skills (Farida et al. 2022; Rizki and Suprapto 2024). In the experimental setting, students participated in collaborative projects and problem-based tasks, applying knowledge creatively and interactively. As a result, the understanding of complex concepts deepened weekly, leading to a consistent upward trend in test performance. The positive progression in scores showed that STEM-based instruction, with a focus on inquiry and exploration, effectively enhanced students’ achievement over time (Kong and Mohd Matore 2022; Wahyu et al. 2020).

The control classroom followed a more traditional teacher-directed curriculum, focusing on content delivery through lectures and standard exercises. Although students in this setting showed a gradual improvement in weekly scores, the progress was less marked compared to the experimental group. Chan (2013) suggested that traditional teaching methods limited the development of creative and critical thinking in students, as it does not motivate inquiry or active problem solving. The inability to offer interactive and applied learning experiences in a STEM classroom led to the slow progress of the control group, outlining the potential limitations of conventional methods in fostering deeper understanding and participation. Traditional teaching methods, which often focused on rote memorization and passive learning, failed to adequately stimulate critical thinking or promote the long-term retention of complex concepts. However, STEM-based learning experiences, which motivated hands-on activities and real-world applications, facilitated active problem solving and collaboration. These methods effectively helped students develop academic skills, including essential competencies such as creativity, adaptability, and communication, crucial in today’s rapidly changing world.

The results of ANOVA clearly proved that STEM-based teaching was more effective in improving students’ performance compared to the traditional teaching method. Meanwhile, both groups started with comparable performance levels, with the STEM group showing a consistent upward trend in the weekly test scores, particularly in the later weeks of the research. The early significant difference in Week 1 suggested that the initial exposure to STEM methods provided an immediate advantage, possibly due to the engaging and hands-on nature of STEM learning (Yannier et al. 2020). The temporary nonsignificant difference in Week 2 reflected an adjustment period for students adapting to the new instructional methods. However, from week 3 onwards, the STEM group showed a marked improvement, reinforcing results from previous research that it fostered deeper conceptual understanding and problem-solving skills (Priemer et al. 2020). The most pronounced differences in weeks 4 and 5 outlined the cumulative benefits of the STEM method, in line with research that outlined how active learning and interdisciplinary problem solving enhanced long-term retention and CT (Kwon et al. 2021). These results strongly supported the argument that STEM-based instruction was more effective than traditional teaching in promoting sustained academic growth, particularly when implemented over a longer period.

The results were in line with previous research in the field. Suherman et al. (2021), reported that STEM-based education significantly strengthened students’ creativity. Similarly, Budhi Akbar (2022), Richardo et al. (2023), and Tikva and Tambouris (2023) stated that positive attitudes toward CT, including social cooperation, confidence, and motivation, significantly enhanced students’ CT and problem solving skills, showing the need to cultivate these attitudes alongside cognitive skills in educational settings. However, research by Sungur and Tekkaya (2006) stated that traditional teaching was heavily textbook-focused and less interactive. A lack of positive attitudes caused well-designed STEM programs to risk fostering disengagement and superficial learning, as this may affect the confidence or collaborative essence essential for innovative contributions. Moreover, a favorable attitude towards CT supported the development of critical competencies relevant for success in modern careers and teaching-based games (Leonard et al. 2018). As technology continues to evolve rapidly, the ability to think computationally becomes increasingly important in various fields. Positive attitudes towards CT contributed to inclusion in education because, when students feel motivated and confident in respective skills, there is a high tendency to actively participate in collaborative learning experiences.

Participants in STEM-based research reported that the method offered a more effective and realistic approach, connecting classroom learning to real-world contexts in a way that traditional methods often failed to achieve. The hands-on, problem-solving nature of STEM education allowed students to engage directly with complex challenges, fostering critical thinking and creativity. The control group, which followed the standard Indonesian curriculum, focused on creativity and problem solving, in a more traditional framework. The Indonesian curriculum mainly centered on theoretical knowledge and didactic learning, with less focus on interdisciplinary applications, while motivating students to develop creative and problem-solving skills. In STEM education, students actively engaged in real-world scenarios, integrating science, technology, engineering, and mathematics to solve practical problems, which created a deeper understanding of how concepts are connected. According to Acar et al. (2018), developing critical and creative thinking in the STEM framework included specific stages, namely, idea generation and problem solving. These stages motivated the analysis of questions posed by the teacher, collaboration with peers to gather insights, as well as discussing and addressing the challenges laid out in the lesson, fostering a more active inquiry-based learning environment.

SEM analysis exhibited strong relationships between students’ attitudes towards STEM, creativity, and CT. This reinforced the idea that an engaging learning environment fosters both creative and analytical skills. Prior research has shown that positive perceptions of STEM education were related to higher motivation and improved problem-solving skills (Chiang et al. 2022; Conradty and Bogner 2019), which enhanced learning outcomes. The model exhibited an acceptable overall fit, with key indices meeting the recommended standards, although the RMSEA value was slightly greater than the ideal threshold. An RMSEA of 0.08 was generally considered a strong fit, with values between 0.05 and 0.08 deemed acceptable, those ranging from 0.08 to 0.10 depicted a marginal fit, and anything exceeding 0.10 was regarded as poor (Fabrigar et al. 1999). However, it is important to contextualize this result. RMSEA values were sensitive to sample characteristics, and a marginally acceptable fit could be theoretically meaningful if supported by other goodness-of-fit indices (e.g., CFI, TLI) and substantive interpretability. Therefore, rather than dismissing the model outright, it was considered whether the marginal fit arose from plausible limitations (e.g., complex constructs, measurement error) or if the modifications could enhance model performance without compromising theoretical integrity. An RMSEA of 0.08 warrants careful consideration and does not necessarily invalidate the model, particularly if it shows reasonable explanatory power and supports established theoretical frameworks. Future research could test an alternative model, for example, adding a direct path from workload to turnover intention to determine if the fit improved meaningfully while retaining theoretical coherence.

The analysis showed students’ attitudes toward STEM were positively associated with both creativity and CT. This was in line with research that outlined the importance of fostering positive perceptions of STEM learning to develop innovative, creative, and CT skills (Chen and Chen 2021; Jiang et al. 2024; Sırakaya et al. 2020). Additionally, creativity played an important mediating role, reinforcing the results that it was integral to CT as well as crucial to students’ ability to apply knowledge in real-world contexts (Hsu et al. 2018). These results offered valuable information on the impact of STEM methods and the enhancement of measurement tools or the incorporation of additional factors, such as teacher influence and classroom environment, improved the precision of the model, further strengthening the inferences drawn.

## 5. Limitations and Future Research

This research provided valuable information on the impact of STEM-based education on students’ creative and CT skills; however, several limitations warrant consideration. For example, the sample size was restricted to a specific geographic area in Indonesia, which could affect the generalizability of the results to other contexts or populations. A quasi-experimental design was adopted, which, despite being practical in educational settings, inherently lacked the rigor of randomized controlled trials. The absence of random assignment to treatment and control groups introduced the possibility of selection bias, as pre-existing differences between groups would have influenced the outcomes rather than the intervention. This compromised the internal validity of the research, making it more difficult to attribute observed effects solely to STEM-based intervention. In addition, uncontrolled extraneous variables, namely, teacher characteristics, students’ motivation, or classroom environment, might have confounded the results. To enhance reliability and validity in future research, the use of randomized designs was recommended. These feasible designs adopted statistical controls and matching techniques to reduce the limitations of quasi-experimental methods. Incorporating longitudinal data also allowed for a deeper understanding of the sustained impact of STEM education on students’ cognitive and attitudinal development over time. Based on this perspective, exploring the specific components of STEM activities that contributed the most significantly to students’ outcomes provided deeper insight into effective teaching practices. These tests were used rather than the maximum performance analyses, which limited the depth of the measurement. The typical performance tests reflected more general attitudes and behaviors, rather than the maximum potential of an individual. Future research should explore the use of maximum performance tests, which can effectively assess the full range of students’ skills and responses, rather than just typical patterns of behavior. Lastly, investigating the role of teacher training and support in implementing STEM curricula would be valuable in understanding how to maximize the benefits of STEM education in different educational settings.

## 6. Conclusions

In conclusion, a quasi-experimental post-test-only design was adopted to investigate the effects of STEM-based teaching methods on students’ creative and CT skills. The results showed a significant positive impact of STEM education on students’ performance, as evidenced by the marked increase in test scores of the experimental group over the intervention period compared to the control group, which adhered to a traditional curriculum. The weekly evaluations depicted that the experimental group showed consistent improvement, while the control group exhibited a more gradual improvement in scores. SEM analysis disclosed strong associations between students’ attitudes towards STEM, creativity, and CT, with creativity identified as a significant mediator in this relationship. These results were in line with the existing literature, which outlined the benefits of STEM education in fostering creative and CT skills, as well as pointing to the limitations of traditional instructional methods in promoting deeper engagement and understanding among students.

This research offered practical insights into the effectiveness of STEM-based teaching methods and also contributed to theoretical understanding in the areas of creativity and CT in education. Practically, the results suggested that integrating STEM approaches in the classroom significantly improved students’ creative thinking and CT skills, regarded as critical competencies in the 21st century. The results offered valuable implications for educators and curriculum designers, focusing on the need for the active incorporation of STEM methods to foster students’ skills that conform to modern educational and industrial demands.

Theoretically, this research advanced the understanding of how STEM education influences cognitive and creative development. By identifying creativity as a significant mediator between attitudes toward STEM learning and CT skills, a new framework was formulated to understand the complex relationship between students’ perceptions of STEM and academic performance. This insight contributed to the growing body of the literature on the cognitive benefits of STEM education, outlining the significant role of creativity in facilitating learning and problem solving. Additionally, the use of SEM provided a robust statistical approach to understanding the interrelations, setting a precedent for future research in this field.

## Figures and Tables

**Table 1:** The demographics of participants.

Demographics		Experiment		Control	
		Frequency	Percentage (%)	Frequency	Percentage (%)
Gender	Female	22	28.57	20	25.97
	Male	17	22.08	18	23.38
School-type	Private	21	27.27	19	24.68
	Public	21	27.27	16	20.78
School place	City	30	38.96	30	38.96
	Suburb	8	10.39	9	11.69
Age	12 years old	14	18.18	15	19.48
	13 years old	21	27.27	21	27.27
	14 years old	3	3.90	3	3.90
Ethnicity	Lampung	5	6.49	4	5.19
	Java	29	37.66	26	33.77
	Sunda	3	3.90	6	7.79
	Batak	1	1.30	0	0.00
	Padang	1	1.30	2	2.60

**Table 2:** STEM activities.

Activity Topic	STEM Focus	Themes	Example Activities
Identifying Benefits of Energy Transformation	Science, Technology, and Mathematics	Energy transformation, daily life applications	Activity 1: Investigate different forms of energy transformation (e.g., mechanical to kinetic, chemical to thermal). In addition, present the results in a report or creative project. Activity 2: Build a simple windmill to show energy conversion.
Wind-Powered Parachute Toy	Science, Technology, Engineering, Mathematics	Engineering design, force and motion, renewable energy	Activity 1: Design and build a wind-powered parachute toy using STEM principles. Wind turbines should be used to generate power. Activity 2: Apply knowledge of physics and design an experiment to measure the flight distance and height of the toy.
Calculating Areas of Planar Shapes	Mathematics, Engineering	Geometry, surface area calculations	Activity 1: Calculate the surface area and dimensions of rectangular, square, and triangular materials to fabricate a parachute model. Activity 2: Measurements of different materials used to determine the most effective for the parachute.
Constructing a Wind-Powered Parachute Toy	Science, Technology, Engineering, Mathematics	Engineering design, practical application of geometric concepts	Activity 1: Cut and prepare materials for the parachute model (rectangles, triangles), then assemble it with a wind-powered rotor. Activity 2: Apply knowledge of basic geometry to build parachute shapes and understand its interaction with the wind.
Reporting on Daily Energy Transformations	Science	Real-world energy conversions	Activity 1: Observe energy transformations in daily appliances (e.g., from electrical to kinetic in a blender). Activity 2: Create an infographic or video report outlining different energy transformations observed in daily activities.

**Table 3:** Loading factor, reliability, and validity of the data.

Variable	Outer Loading	Cronbach’s Alpha	Composite Reliability (rho_c)	AVE	Skewness	Kurtosis
Computational thinking (CT)		0.821	0.881	0.651	−0.15	0.34
CT1	0.861					
CT2	0.862					
CT3	0.796					
CT4	0.699					
Creative thinking (CR)		0.777	0.852	0.593	0.09	−0.29
Ela	0.729					
Flu	0.815					
Flx	0.874					
Ori	0.643					
Attitude towards STEM (ST)		0.920	0.943	0.806	−0.05	−0.07
ST1	0.907					
ST2	0.907					
ST3	0.890					
ST4	0.887					

**Table 4:** Discriminant validity by HTMT.

	CR	CT	ST
CR	-		
CT	0.758	-	
ST	0.537	0.848	-

**Table 5:** Weekly evaluation in experimental and control classes.

Number of Tasks	Teaching Methods	Weekly Test				
		Week 1 *M* (*SD*)	Week 2 *M* (*SD*)	Week 3 *M* (*SD*)	Week 4 *M* (*SD*)	Week 5 *M* (*SD*)
1	STEM	47.22 (1.94)	51.21 (1.56)	52.96 (1.74)	72.30 (1.35)	76.20 (1.50)
2		45.62 (0.60)	48.61 (0.47)	53.85 (0.40)	71.48 (0.68)	76.21 (0.74)
3		46.32 (0.23)	49.81 (0.15)	54.85 (0.11)	71.55 (0.13)	75.66 (0.08)
4		51.43 (0.08)	51.99 (0.07)	54.70 (0.06)	71.60 (0.06)	76.34 (0.05)
5		51.44 (0.05)	52.68 (0.04)	54.75 (0.02)	73.30 (0.03)	76.65 (0.02)
6		52.21 (0.04)	55.53 (0.03)	56.33 (0.03)	70.31 (0.02)	77.41 (0.02)
1	Control	41.22 (0.04)	44.65 (0.03)	51.55 (0.02)	65.20 (0.02)	66.70 (0.02)
2		42.44 (0.03)	46.55 (0.02)	52.75 (0.02)	64.25 (0.01)	66.80 (0.01)
3		43.20 (0.03)	48.44 (0.02)	52.65 (0.01)	63.21 (0.01)	64.35 (0.01)
4		44.50 (0.02)	52.35 (0.01)	53.45 (0.01)	62.54 (0.01)	64.30 (0.00)
5		45.87 (0.01)	49.80 (0.01)	52.27 (0.01)	61.55 (0.01)	64.20 (0.01)
6		48.93 (0.01)	48.92 (0.01)	53.21 (0.01)	66.45 (0.01)	66.20 (0.01)

**Table 6:** Statistics descriptive between variables.

Variable		*M*	*SD*	*Min*	*Max*	CR	CT	ST
Control	CR	3.17	0.64	2.00	4.25	1		
	CT	3.17	0.77	1.00	5.00	0.654 **	1	
	ST	3.30	0.93	1.00	5.00	0.450 **	0.732 **	1
STEM	CR	3.06	0.72	1.75	5.00	1		
	CT	3.05	0.85	1.00	5.00	0.576 **	1	
	ST	3.15	1.12	1.00	5.00	0.457 **	0.742 **	1

Note: ** Correlation is significant at the 0.01 level (2-tailed). CR = creative thinking; CT = computational thinking; and ST = STEM attitude.

**Table 7:** Bootstrapping of the variables.

Path	Original Sample (O)	Sample Mean (M)	STDEV	T Statistics (|O/STDEV|)	STDEV Residual	*p*	2.5%	97.5%
CR -> CT	0.363	0.374	0.089	4.084	−0.12	<.001	0.198	0.547
STEM -> CR	0.493	0.508	0.080	6.177	−0.19	<.001	0.345	0.656
STEM -> CT	0.753	0.757	0.052	14.524	−0.08	<.001	0.388	0.724
ST -> CR -> CT	0.179	0.190	0.055	3.232	−0.20	<.001	0.091	0.307

## Data Availability

The data presented in this study are available on request from the corresponding author.

## References

[B1-jintelligence-13-00088] Acar Dilber, Tertemiz Neşe, Taşdemir Adem (2018). The Effects of STEM Training on the Academic Achievement of 4th Graders in Science and Mathematics and Their Views on STEM Training. International Electronic Journal of Elementary Education.

[B2-jintelligence-13-00088] Aguilera David, Ortiz-Revilla Jairo (2021). STEM vs. STEAM Education and Student Creativity: A Systematic Literature Review. Education Sciences.

[B3-jintelligence-13-00088] Angeli Charoula, Voogt Joke, Fluck Andrew, Webb Mary, Cox Margaret, Malyn-Smith Joyce, Zagami Jason (2016). A K-6 Computational Thinking Curriculum Framework: Implications for Teacher Knowledge. Journal of Educational Technology & Society.

[B4-jintelligence-13-00088] Astawan I. Gede, Suarjana I. Made, Werang Basilius, Asaloei Sandra Ingried, Sianturi Murni, Elele Emmanuel Chinedu (2023). STEM-Based Scientific Learning and Its Impact on Students’ Critical and Creative Thinking Skills: An Empirical Study. Jurnal Pendidikan IPA Indonesia.

[B5-jintelligence-13-00088] Baran Jovanovic Evrim, Bilici SEDEF Canbazoğlu, Mesutoğlu Canan, Ocak Ceren (2019). The Impact of an Out-of-school STEM Education Program on Students’ Attitudes toward STEM and STEM Careers. School Science and Mathematics Journal for All Science And Mathematics Teachers.

[B6-jintelligence-13-00088] Beghetto Ronald A., Kaufman James C. (2014). Classroom Contexts for Creativity. High Ability Studies.

[B7-jintelligence-13-00088] Besançon Maud, Lubart Todd (2008). Differences in the Development of Creative Competencies in Children Schooled in Diverse Learning Environments. Learning and Individual Differences.

[B8-jintelligence-13-00088] Boeve-De Pauw Jelle, De Loof Haydée, Walan Susanne, Gericke Niklas, Van Petegem Peter (2024). Teachers’ Self-Efficacy and Role When Teaching STEM in High-Tech Informal Learning Environments. Research in Science & Technological Education.

[B9-jintelligence-13-00088] Borg Preca Christabel, Baldacchino Leonie, Briguglio Marie, Mangion Margaret (2023). Are STEM Students Creative Thinkers?. Journal of Intelligence.

[B10-jintelligence-13-00088] Budhi Akbar Elsa (2022). Correlation Between Social Attitude and Computational Thinking Ability. Journal of Positive School Psychology.

[B11-jintelligence-13-00088] Chan Zenobia Cy (2013). Exploring Creativity and Critical Thinking in Traditional and Innovative Problem-based Learning Groups. Journal of Clinical Nursing.

[B12-jintelligence-13-00088] Chen Chen, Hardjo Stephanie, Sonnert Gerhard, Hui Jiaojiao, Sadler Philip M. (2023). The Role of Media in Influencing Students’ STEM Career Interest. International Journal of STEM Education.

[B13-jintelligence-13-00088] Chen Kieranna, Chen Chenin (2021). Effects of STEM Inquiry Method on Learning Attitude and Creativity. Eurasia Journal of Mathematics, Science and Technology Education.

[B14-jintelligence-13-00088] Cheng Li, Wang Xiaoman, Ritzhaupt Albert D. (2023). The Effects of Computational Thinking Integration in STEM on Students’ Learning Performance in K-12 Education: A Meta-Analysis. Journal of Educational Computing Research.

[B15-jintelligence-13-00088] Chiang Feng-Kuang, Zhang Yicong, Zhu Dan, Shang Xiaojing, Jiang Zhujun (2022). The Influence of Online STEM Education Camps on Students’ Self-Efficacy, Computational Thinking, and Task Value. Journal of Science Education and Technology.

[B16-jintelligence-13-00088] Conradty Cathérine, Bogner Franz X. (2019). From STEM to STEAM: Cracking the Code? How Creativity & Motivation Interacts with Inquiry-Based Learning. Creativity Research Journal.

[B17-jintelligence-13-00088] Cromwell Johnathan R., Haase Jennifer, Vladova Gergana (2023). The Creative Thinking Profile: Predicting Intrinsic Motivation Based on Preferences for Different Creative Thinking Styles. Personality and Individual Differences.

[B18-jintelligence-13-00088] De Jager Cherylene, Muller Anton, Roodt Gert (2013). Developing Creative and Innovative Thinking and Problem-Solving Skills in a Financial Services Organisation. SA Journal of Human Resource Management.

[B19-jintelligence-13-00088] Ernst Jeremy, Williams Thomas, Clark Aaron, Kelly Daniel, Sutton Kevin (2018). K-12 STEM Educator Autonomy: An Investigation of School Influence and Classroom Control. Journal of STEM Education.

[B20-jintelligence-13-00088] Fabrigar Leandre R., Wegener Duane T., MacCallum Robert C., Strahan Erin J. (1999). Evaluating the Use of Exploratory Factor Analysis in Psychological Research. Psychological Methods.

[B21-jintelligence-13-00088] Farida Farida, Supriadi Nanang, Andriani Siska, Pratiwi Dona Dinda, Suherman Suherman, Muhammad Rosida Rakhmawati (2022). STEM Approach and Computer Science Impact the Metaphorical Thinking of Indonesian Students’. Revista de Educación a Distancia (RED).

[B22-jintelligence-13-00088] Fessakis Georgios, Prantsoudi Stavroula (2019). Computer Science Teachers’ Perceptions, Beliefs and Attitudes on Computational Thinking in Greece. Informatics in Education.

[B23-jintelligence-13-00088] Fitzakerley Janet L., Michlin Michael L., Paton John, Dubinsky Janet M. (2013). Neuroscientists’ Classroom Visits Positively Impact Student Attitudes. PLoS ONE.

[B25-jintelligence-13-00088] Fornell Claes, Larcker David F. (1981). Evaluating Structural Equation Models with Unobservable Variables and Measurement Error. Journal of Marketing Research.

[B26-jintelligence-13-00088] Galanti Terrie M., Holincheck Nancy M. (2024). Integrating Computational Thinking in Elementary Using the Engineering Design Process. School Science and Mathematics.

[B27-jintelligence-13-00088] Goos Merrilyn, Carreira Susana, Namukasa Immaculate Kizito (2023). Mathematics and Interdisciplinary STEM Education: Recent Developments and Future Directions. ZDM—Mathematics Education.

[B28-jintelligence-13-00088] Guilford Joy Paul (1950). Fundamental Statistics in Psychology and Education.

[B29-jintelligence-13-00088] Guzey S. Selcen, Harwell Michael, Moore Tamara (2014). Development of an Instrument to Assess Attitudes toward Science, Technology, Engineering, and Mathematics (STEM). School Science and Mathematics.

[B30-jintelligence-13-00088] Hamutoğlu Nazire Burçin, Başarmak Uğur, Çam Emre, Salar Hurşit Cem (2022). Investigation of Secondary School Students’ Attitudes Towards Computational Thinking, Problem-Solving Skills and Research-Inquiry. Türk Akademik Yayınlar Dergisi (TAY Journal).

[B31-jintelligence-13-00088] Henriksen Danah, Mishra Punya, Fisser Petra (2016). Infusing Creativity and Technology in 21st Century Education: A Systemic View for Change. Educational Technology & Society.

[B32-jintelligence-13-00088] Hershkovitz Arnon, Sitman Raquel, Israel-Fishelson Rotem, Eguíluz Andoni, Garaizar Pablo, Guenaga Mariluz (2019). Creativity in the Acquisition of Computational Thinking. Interactive Learning Environments.

[B33-jintelligence-13-00088] Hsu Ting-Chia, Chang Shao-Chen, Hung Yu-Ting (2018). How to Learn and How to Teach Computational Thinking: Suggestions Based on a Review of the Literature. Computers & Education.

[B34-jintelligence-13-00088] Hu Li-tze, Bentler Peter M. (1999). Cutoff Criteria for Fit Indexes in Covariance Structure Analysis: Conventional Criteria versus New Alternatives. Structural Equation Modeling: A Multidisciplinary Journal.

[B35-jintelligence-13-00088] Huda Syamsul, Rinaldi Achi, Suherman Suherman, Sugiharta Iip, Astuti Dian Widi, Fatimah Okis, Prasetiyo Andika Eko (2019). Understanding of Mathematical Concepts in the Linear Equation with Two Variables: Impact of E-Learning and Blended Learning Using Google Classroom. Al-Jabar: Jurnal Pendidikan Matematika.

[B36-jintelligence-13-00088] Jiang Haozhe, Islam A. Y. M. Atiquil, Gu Xiaoqing, Guan Jia (2024). How Do Thinking Styles and STEM Attitudes Have Effects on Computational Thinking? A Structural Equation Modeling Analysis. Journal of Research in Science Teaching.

[B37-jintelligence-13-00088] Kelley Todd R., Knowles John Geoff (2016). A Conceptual Framework for Integrated STEM Education. International Journal of STEM Education.

[B38-jintelligence-13-00088] Kline Rex B. (2015). Principles and Practice of Structural Equation Modeling.

[B39-jintelligence-13-00088] Kong Suik Fern, Mohd Matore Mohd Effendi Ewan (2022). Can a Science, Technology, Engineering, and Mathematics (STEM) Approach Enhance Students’ Mathematics Performance?. Sustainability.

[B40-jintelligence-13-00088] Korkmaz Özgen, Çakir Recep, Özden Muhammet Yaşar (2017). A Validity and Reliability Study of the Computational Thinking Scales (CTS). Computers in Human Behavior.

[B41-jintelligence-13-00088] Kwon Kyungbin, Ottenbreit-Leftwich Anne T., Brush Thomas A., Jeon Minji, Yan Ge (2021). Integration of Problem-Based Learning in Elementary Computer Science Education: Effects on Computational Thinking and Attitudes. Educational Technology Research and Development.

[B42-jintelligence-13-00088] Kwong-Kay Wong Ken (2013). Partial Least Squares Structural Equation Modeling (PLS-SEM) Techniques Using SmartPLS. Marketing Bulletin.

[B43-jintelligence-13-00088] Lee Irene, Malyn-Smith Joyce (2020). Computational Thinking Integration Patterns along the Framework Defining Computational Thinking from a Disciplinary Perspective. Journal of Science Education and Technology.

[B44-jintelligence-13-00088] Leonard Jacqueline, Mitchell Monica, Barnes-Johnson Joy, Unertl Adrienne, Outka-Hill Jill, Robinson Roland, Hester-Croff Carla (2018). Preparing Teachers to Engage Rural Students in Computational Thinking Through Robotics, Game Design, and Culturally Responsive Teaching. Journal of Teacher Education.

[B45-jintelligence-13-00088] Lesseig Kristin, Nelson Tamara Holmlund, Slavit David, Seidel Ryan August (2016). Supporting Middle School Teachers’ Implementation of STEM Design Challenges. School Science and Mathematics.

[B46-jintelligence-13-00088] Lin Kuen-Yi, Yeh Yi-Fen, Hsu Ying-Shao, Wu Jen-Yi, Yang Kai-Lin, Wu Hsin-Kai (2023). STEM Education Goals in the Twenty-First Century: Teachers’ Perceptions and Experiences. International Journal of Technology and Design Education.

[B47-jintelligence-13-00088] Lo Chung Kwan (2021). Design Principles for Effective Teacher Professional Development in Integrated STEM Education. Educational Technology & Society.

[B48-jintelligence-13-00088] Lucas Bill, Claxton Guy, Spencer Ellen (2013). Progression in Student Creativity in School: First Steps towards New Forms of Formative Assessments. OECD Education Working Papers.

[B49-jintelligence-13-00088] Makhmasi Sohailah, Zaki Rachad, Barada Hassan, Al-Hammadi Yousof (2012). Factors Influencing STEM Teachers’ Effectiveness in the UAE. Paper presented at 2012 Frontiers in Education Conference Proceedings.

[B50-jintelligence-13-00088] Maskur Ruhban, Suherman Suherman, Andari Tri, Anggoro Bambang Sri, Muhammad Rosida Rakhmawati, Untari Erny (2022). La Comparación Del Enfoque STEM y El Modelo de Aprendizaje SSCS Para La Escuela Secundaria Basado En El Plan de Estudios K-13: El Impacto En La Capacidad de Pensamiento Creativo y Crítico. Revista de Educación a Distancia (RED).

[B51-jintelligence-13-00088] Meyers Lawrence S., Gamst Glenn, Guarino Anthony Joseph (2016). Applied Multivariate Research.

[B52-jintelligence-13-00088] Ministry of Education (2014). Kebudayaan, Ilmu Pengetahuan Alam SMP/MTs Untuk Kelas VII Semester 1, Edisi Revisi.

[B53-jintelligence-13-00088] Nouri Jalal, Zhang Lechen, Mannila Linda, Norén Eva (2020). Development of Computational Thinking, Digital Competence and 21st Century Skills When Learning Programming in K-9. Education Inquiry.

[B54-jintelligence-13-00088] OECD (2019). Draft Framework for the Assessment of Creative Thinking in PISA 2021.

[B55-jintelligence-13-00088] Ouyang Fan, Xu Weiqi (2024). The Effects of Educational Robotics in STEM Education: A Multilevel Meta-Analysis. International Journal of STEM Education.

[B56-jintelligence-13-00088] Pont-Niclòs Isabel, Martín-Ezpeleta Antonio, Echegoyen-Sanz Yolanda (2024). Scientific Creativity in Secondary Students and Its Relationship with STEM-Related Attitudes, Engagement and Work Intentions. Frontiers in Education.

[B57-jintelligence-13-00088] Priemer Burkhard, Eilerts Katja, Filler Andreas, Pinkwart Niels, Rösken-Winter Bettina, Tiemann Rüdiger, Belzen Annette Upmeier Zu (2020). A Framework to Foster Problem-Solving in STEM and Computing Education. Research in Science & Technological Education.

[B58-jintelligence-13-00088] Psycharis Sarantos, Kotzampasaki Evangelia (2019). The Impact of a STEM Inquiry Game Learning Scenario on Computational Thinking and Computer Self-Confidence. Eurasia Journal of Mathematics, Science and Technology Education.

[B59-jintelligence-13-00088] Richardo Rino, Dwiningrum Siti Irene Astuti, Wijaya Ariyadi, Retnawati Heri, Wahyudi Andi, Sholihah Dyahsih Alin, Hidayah Khasanah Nur (2023). The Impact of STEM Attitudes and Computational Thinking on 21st-Century via Structural Equation Modelling. International Journal of Evaluation and Research in Education.

[B60-jintelligence-13-00088] Rizki Iqbal Ainur, Suprapto Nadi (2024). Project-Oriented Problem-Based Learning through SR-STEM to Foster Students’ Critical Thinking Skills in Renewable Energy Material. Journal of Science Education and Technology.

[B61-jintelligence-13-00088] Sawyer John (2012). Sacred Languages and Sacred Texts.

[B62-jintelligence-13-00088] Sengupta Pratim, Kinnebrew John S., Basu Satabdi, Biswas Gautam, Clark Douglas (2013). Integrating Computational Thinking with K-12 Science Education Using Agent-Based Computation: A Theoretical Framework. Education and Information Technologies.

[B63-jintelligence-13-00088] Shahbazloo Fatemeh, Mirzaie Rasol Abdullah (2023). Investigating the Effect of 5E-Based STEM Education in Solar Energy Context on Creativity and Academic Achievement of Female Junior High School Students. Thinking Skills and Creativity.

[B64-jintelligence-13-00088] Sherin Bruce, diSessa Andrea A., Hammer David (1993). Dynaturtle Revisited: Learning Physics Through Collaborative Design of a Computer Model. Interactive Learning Environments.

[B65-jintelligence-13-00088] Sırakaya Mustafa, Sırakaya Didem Alsancak, Korkmaz Özgen (2020). The Impact of STEM Attitude and Thinking Style on Computational Thinking Determined via Structural Equation Modeling. Journal of Science Education and Technology.

[B66-jintelligence-13-00088] Stoet Gijsbert, Geary David C. (2018). The Gender-Equality Paradox in Science, Technology, Engineering, and Mathematics Education. Psychological Science.

[B67-jintelligence-13-00088] Struyf Annemie, De Loof Haydée, Boeve-de Pauw Jelle, Van Petegem Peter (2019). Students’ Engagement in Different STEM Learning Environments: Integrated STEM Education as Promising Practice?. International Journal of Science Education.

[B68-jintelligence-13-00088] Suherman Suherman, Vidákovich Tibor (2022). Assessment of Mathematical Creative Thinking: A Systematic Review. Thinking Skills and Creativity.

[B69-jintelligence-13-00088] Suherman Suherman, Vidákovich Tibor (2024a). Mathematical Creative Thinking-Ethnomathematics Based Test: Role of Attitude toward Mathematics, Creative Style, Ethnic Identity, and Parents’ Educational Level. Revista de Educación a Distancia (RED).

[B70-jintelligence-13-00088] Suherman Suherman, Vidákovich Tibor (2024b). Role of Creative Self-Efficacy and Perceived Creativity as Predictors of Mathematical Creative Thinking: Mediating Role of Computational Thinking. Thinking Skills and Creativity.

[B71-jintelligence-13-00088] Suherman Suherman, Vidákovich Tibor, Komarudin Komarudin (2021). STEM-E: Fostering Mathematical Creative Thinking Ability in the 21st Century. Journal of Physics: Conference Series.

[B72-jintelligence-13-00088] Sun Lihui, Hu Linlin, Yang Weipeng, Zhou Danhua, Wang Xiaoqian (2021). STEM Learning Attitude Predicts Computational Thinking Skills among Primary School Students. Journal of Computer Assisted Learning.

[B73-jintelligence-13-00088] Sungur Semra, Tekkaya Ceren (2006). Effects of Problem-Based Learning and Traditional Instruction on Self-Regulated Learning. The Journal of Educational Research.

[B74-jintelligence-13-00088] Tan Aik-Ling, Ong Yann Shiou, Ng Yong Sim, Tan Jared Hong Jie (2023). STEM Problem Solving: Inquiry, Concepts, and Reasoning. Science & Education.

[B75-jintelligence-13-00088] Tikva Christina, Tambouris Efthimios (2023). The Effect of Scaffolding Programming Games and Attitudes towards Programming on the Development of Computational Thinking. Education and Information Technologies.

[B76-jintelligence-13-00088] Torrance Ellis Paul (1974). Torrance Tests of Creative Thinking: Norms-Technical Manual.

[B24-jintelligence-13-00088] US STEM Task Force (2014). Innovate: A Blueprint for Science, Technology, Engineering, and Mathematics in California Public Education.

[B77-jintelligence-13-00088] Verawati NNSP, Rijal Khaerul, Grendis Nuraqilla Waidha B. (2023). Examining STEM Students’ Computational Thinking Skills through Interactive Practicum Utilizing Technology. International Journal of Essential Competencies in Education.

[B78-jintelligence-13-00088] Wahyu Yuliana, Suastra I. Wayan, Sadia I. Wayan, Suarni Ni Ketut (2020). The Effectiveness of Mobile Augmented Reality Assisted Stem-Based Learning on Scientific Literacy and Students’ Achievement. International Journal of Instruction.

[B79-jintelligence-13-00088] Wang Bin, Li Ping-ping (2022). Digital Creativity in STEM Education: The Impact of Digital Tools and Pedagogical Learning Models on the Students’ Creative Thinking Skills Development. Interactive Learning Environments.

[B80-jintelligence-13-00088] Wang Feng, Huang Jun, Zheng Xiao-Li, Wu Jun-Qi, Zhao An-Ping (2024). STEM Activities for Boosting Pupils’ Computational Thinking and Reducing Their Cognitive Load: Roles of Argumentation Scaffolding and Mental Rotation. Journal of Research on Technology in Education.

[B81-jintelligence-13-00088] Weintrop David, Beheshti Elham, Horn Michael, Orton Kai, Jona Kemi, Trouille Laura, Wilensky Uri (2016). Defining Computational Thinking for Mathematics and Science Classrooms. Journal of Science Education and Technology.

[B82-jintelligence-13-00088] Wing Jeannette M. (2006). Computational Thinking. Communications of the ACM.

[B83-jintelligence-13-00088] Wiswall Matthew, Stiefel Leanna, Schwartz Amy Ellen, Boccardo Jessica (2014). Does Attending a STEM High School Improve Student Performance? Evidence from New York City. Economics of Education Review.

[B84-jintelligence-13-00088] Xu Weiqi, Ouyang Fan (2022). The Application of AI Technologies in STEM Education: A Systematic Review from 2011 to 2021. International Journal of STEM Education.

[B85-jintelligence-13-00088] Xu Weiqi, Geng Fengji, Wang Lin (2022). Relations of Computational Thinking to Reasoning Ability and Creative Thinking in Young Children: Mediating Role of Arithmetic Fluency. Thinking Skills and Creativity.

[B86-jintelligence-13-00088] Yadav Aman, Caeli Elisa Nadire, Ocak Ceren, Macann Victoria (2022). Teacher Education and Computational Thinking: Measuring Pre-Service Teacher Conceptions and Attitudes. Paper presented at 27th ACM Conference on on Innovation and Technology in Computer Science Education.

[B87-jintelligence-13-00088] Yannier Nesra, Hudson Scott E., Koedinger Kenneth R. (2020). Active Learning Is About More Than Hands-On: A Mixed-Reality AI System to Support STEM Education. International Journal of Artificial Intelligence in Education.

[B88-jintelligence-13-00088] Yasin Muhamad, Huda Syamsul, Septiana Reni, Palupi Endah Kinarya (2020). Mathematical Critical Thinking Ability: The Effect of Scramble Learning Model Assisted by Prezi in Islamic School. Journal of Physics: Conference Series.

